# Selections and Abstracts

**Published:** 1915-02

**Authors:** 


### SELECTIONS AND ABSTRACTS

Dr. Dudley S. Reynolds, one of the best known physicians of Louisville, died suddenly of heart disease at his country home, "West Meath," near Pleasure Ridge Park. He was 72 years old. He had been ill since Christmas of the grip, but his condition at no time was considered critical. He was seized with a heart attack at 11 o'clock after he had gone to bed. Dr. Wade Shacklette, of Pleasure Ridge Park, was called, but Dr. Reynolds was dead when he arrived.

Dr. Reynolds had practiced his profession in Louisville half a century. He was studying medicine when the Civil War broke out and left his studies to become an assistant surgeon in the Union army. At the end of the struggle he returned to this city and completed his college course.

Born in Glasgow, Ky., on August 31, 1842, Dr. Reynolds, who was the son of the Rev. Thomas Reynolds and Mary Nichols Reynolds, received his public school education there and at the Trimble High School in Trimble County. At an early age he came to Louisville and entered the university, but deserted his studies a year later at the outbreak of the Civil War. He served as assistant surgeon in the Union army until the end of the war, and then returned here to resume his college work.

After receiving his degree Dr. Reynolds took post-graduate courses at Johns Hopkins Hospital and the University of Chicago and in foreign cities, including London, Berlin, Vienna and Paris.

Dr. Reynolds was a member of the American, Southern, Kentucky, Ohio and the Mississippi Valley Medical Associations and the Jefferson County Medical Society. Of practically all of these he has been elected president. He also was an inmerged with the University of Louisville in 1907.
structor at the Old Hospital College of Medicine, which was

Dr. Reynolds had been married three times. About eight years ago he married Miss Lily B. Baldwin, who survives him. He also leaves a son, Bruce Reynolds, and a daughter, Mrs. Elizabeth Werlein, both of Cincinnati; three grandchildren and a sister, Mrs. J. Reynolds, of Glasgow.

---

### THE HARRISON ACT.

In two days from this date the Harrison Act—the national anti-narcotic law—goes into effect, and at this writing the greatest confusion exists in the profession as to its duties and its rights under the Act. It is probably too late to call attention to the fact that the passage of a national law of this kind was not warranted by the conditions that existed. The excessive use of narcotic drugs is not universally existent over the whole United States. It is undoubtedly confined almost entirely to the red-light districts of cities and to a few rural communities here and there that are supplied with such low-grade (educationally) physicians, that these latter dull the discomforts or pains of their patients with hypodermics of morphia because they know not what else to do. Sporadic cases, of course, occur widely scattered among the neurasthenics and neurotics of one kind or another, but these are just as likely to occur under the Harrison Act as if there were no such ill-considered piece of legislation in existence.

That the total number of drug users in the United States has been grossly exaggerated by the advocates of this law in order to insure its passage by Congress is evidenced by the statements used by them in the case of the State of Ohio. It was asserted that there were eight thousand drug addicts in the State of Ohio alone, and largely on the strength of this statement the Ohio Legislature passed the anti-narcotic law of last year. If this law was honestly administered and eight thousand drug addicts had been suddenly deprived of their stimulant or narcotic supplies (the morphine addicts using from two to forty grains a day, and the cocaine addicts from five to sixty grains a day), something would have been heard of the havoc wrought in the State of Ohio, for in the persons of the morphinists, at least, we would have had an epidemic of illness that would have been State-wide and have overshadowed all outbreaks of typhoid, scarlet fever, measles or what-not that are deemed worthy of record in the public press. In anticipation of such a state of affairs, it was proposed to use the Lima State Hospital as a refuge for these cases, but it presently turned out that they were even more mythical than the criminal insane for whom the hospital was built.

It is not of record that the number of these cases has increased in the municipal hospitals of the State, and the medical directors of private sanitaria deny that they have any more cases than before the Ohio law was passed. Inquiry among reputable physicians brings to light but very few cases among the classes that are embraced in their practice, and the conclusion is inevitable that the vice, if it is proper to so stigmatize the habit, is confined almost exclusively to the inhabitants of the underworld and mainly, among them, to the female sex.

The interpretation of the law, even, among those who urged and are responsible for its passage, is a matter of such uncertainty that individual physicians are in doubt as to when and where these valuable records are to be made. Is one exempt from the necessity of record when one dispenses in one's office or when one writes a prescription in the patient's house? Or is it vice versa, or both, or neither? The avowed object of the law was to be the ability on the part of the administering officials to trace the ultimate destination of every grain of opium or cocaine that entered the United States, and yet, as the law now stands, with its exceptions in favor of pharmacists, and patent medicine fakirs, who make nostrums containing the forbidden drugs, and the exceptions in favor of physicians under certain circumstances (if there are any), it will be utterly impossible to make the importations and the ultimate consumption, as it can be discovered, balance by many thousands of pounds.

This law goes upon trial March 1, and the first few months of its intricate existence will undoubtedly be given up to the disentagling knots of procedure and the rectification of innumerable blunders. Certainly out of our experience in the next year or two will come an amended law, which will in reality trace every grain of the forbidden medicaments—stamp out drug addiction even in the underworld and help instead of hinder reputable physicians in the proper practice of their profession.—Lancet Clinic.

---

### EXPERIENCES OF THE NEW YORK HEALTH DEPARTMENT IN TYPHOID IMMUNIZATION.

By L. I. Harris, M. D., Assistant to Director, Bureau of Infectious diseases.

Case Reports by Morris L. Ogan, M. D., Chief, Division of Typhoid Fever, New York.

(Continued.)

A study of the third class of cases assumes special importance, because it raises the question whether it is a safe and proper practice to vaccinate those who are much exposed in an epidemic, or in intimate contact with typhoid fever cases, in the capacity of medical attendant or nurse. Here, too, isolated examples are of no value in helping one to arrive at a judgment of the value of prophylactic treatment, and one must perforce utilize all available records from authoritative sources, and from such cumulative evidence derive one's conclusions.

It was in connection with this type of case that Wright offered his theory of the negative phase, and expressed his disapproval of antityphoid inoculations during the period of its presence. His theory and the arguments in its support were based on laboratory experiments, and when they were first promulgated, many excellent clinicians were persuaded by their apparent validity to oppose such prophylactic treatment during epidemics. A characteristic example is McCrae, who, in his article on "Enteric Fever" in Osler's "System," said, "It is evident that it would not be wise to vaccinate during an epidemic as the procedure apparently makes the individual less resistant to infection for a time."

As has already been stated in a previous section, Leishman in a series of practical tests conducted on 100 individuals exposed at an asylum, made observations that refuted Wright's theoretic views, and in this he was seconded by a goodly number of careful clinicians and laboratory workers. Of the latter, Pfeiffer and Friedbergers' experiments were particularly valuable.

Cullinan, of Dublin, a pupil of Wright, inoculated 500 persons at the Richmond asylum during an epidemic of typhoid fever that lasted five months, and only 1.35 per cent contracted the disease; and those who came down with it were already in the incubation stage. Of the 114 uninoculated nurses, 14.9 per cent developed typhoid fever.

Spooner inoculated 29 out of 65 persons in a small Vermont town, at a time when 17 had already been brought down by an attack. Of these 29 only one person (3.5 per cent) contracted typhoid fever, whereas of 19 who had refused vaccination, 5 developed the disease—26.3 per cent.

Hunt, who in 1912 took charge of the situation in Troy, Pa., where an epidemic was in progress, and who, judged by his report at the time, is not an enthusiast as to antityphoid prophylaxis, found 1,343 persons who had been definitely exposed. Of 761 who accepted vaccination and were well at the time of the first injection, a total of 37 (4.86 per cent) developed typhoid fever. Of these 37 cases, 28 occurred after the first injection, 7 after the second and 2 after the third injection. Contrast with this the 582 who were not vaccinated and among whom 65 (14.28 per cent) came down with enteric fever. Whatever his deductions from the facts observed may have been, his testimony, taken together with that of others, seems strongly to argue for a decided protective action of the vaccine even in the presence of an epidemic. Even his mortality statistics would tend to confirm one's faith in the value of protective inoculations, for, of the 37 cases that appeared among the vaccinated, there was but one death (0.27 per cent) as opposed to 17 (8.85 per cent) among the 65 unvaccinated. Hunt takes issue with Spooner, who believed that there was an acceleration of the onset in the infected vaccinated cases. The former contends that the incubation period was prolonged, and the onset consequently delayed until many days after the second inoculation in four cases, and until seven days after inoculation in another case. The evidence which these particular cases furnish strongly offsets, in Hunt's opinion, the prompt appearance of typhoid fever in 11 cases after the first inoculation.

Nor are these by any means isolated instances in which antityphoid vaccination during epidemics gave an excellent account of itself. It has been recognized that the exposure of hospital attendants, nurses, etc.—particularly nurses who do night duty—predisposes them to typhoid fever, in many cases, quite as much as would an epidemic. Observations made on this group, therefore, are of telling value. Also it should be borne in mind in this connection that nurses are known to be many times more liable to contract the disease than are the others of a city's population; Joslin and Overlander, of Boston, have found that the incidence of this disease is from eight to twelve times greater among them; while Hachtel and Stoner found the rate among this group of workers for five years in Baltimore, 500 per ten thousand nurses and hospital attendants, or from twelve to twenty times greater than in the city generally. Yet at the Massachusetts General Hospital, where immunization was carried out for three years, the morbidity was only 1.4 per cent, or 19 cases in 1,361, even including nurses on night duty. In Baltimore, likewise, the institution of vaccination abolished the disease among the nurses (among 82 nurses during five years who had not been vaccinated, 7 or 8.5 per cent, contracted typhoid fever.)

Ravenel, in a small town in which an epidemic was at its height, vaccinated 116 persons, and not one new case appeared thereafter.

Wright and Leishman vaccinted at Maidstone, when an epidemic had attained its maximum intensity, and with success. At Torrington Conn., of 80 nurses who were employed, 45 accepted vaccination and remained free from the disease, while of 35 who refused the treatment, 3 cases subsequently developed.

Peterson reports that in one house in which the sanitary conditions were of the worst, there were 14 occupants of whom 7 were down with typhoid fever. The remaining 7 were immunized and no further cases developed.

From this summary of statistical data which could be greatly enlarged on, it must appear that the fear of a negative phase, and the withholding of immunization from those closely in contact with typhoid fever cases, is not grounded on sound and logical reasoning. The experiences of scientific and conservative observers combine to prove the merit of this prophylactic procedure, and should commend it as a duty to those charged with the care of the community's health, wherever and whenever typhoid fever appears in its midst.

Moreover, the freedom with which conservative clinicians in constantly increasing numbers employ the vaccine as a therapeutic measure, to moderate the severity of this disease when already established, and to diminish its mortality rate, should serve to dispose of any distrust of its effects, which still lingers in some quarters. Wright believes that through its use, the incidence of typhoid fever is diminished about one-half, and the mortality rate still more. These statements are not to be construed as a support of those who believe in the therapeutic efficacy of the vaccine, for that is still sub judice.

Hornor's studies in the therapeutic employment of the vaccine in 40 of 135 cases, while they show no decided advantage from its use as a curative agent, exhibit, however, no evidence that could in any manner be construed as an argument against the safety of its administration. In addition, the restriced scale on which he has employed it cannot make his experience serve as more than a preliminary experimental note. Compared with Wright's extensive experiences, the limitations of Hornor's report can be better understood.

"

Russell further reports that in the navy, one mild case followed the second injection. Another man at Guantanamo, Cuba, received one dose, and two days later complained of headache, had a temperature of 104.6 and manifested dulness of intellect. In twenty-four hours he became rapidly worse. He died seventy-six hours after admission to hospital, and seven days after vaccination. The necropsy showed that he was at the beginning of the third week of the disease. The infection was one week old when the single dose of vaccine had been administered. Ambulatory cases are notorious for developing severe symptoms late in the course of the disease, and for their high mortality rate.

The case exemplifies strikingly the manifest difficulty of determining in these exceptional instances whether typhoid infection has already gained a foothold, and is like the cases of Mrs. M. and her daughter, Ruth, as recorded in the preceding case reports.

In the Lancet, W. Broughton-Alcock reports the use of living sensitized typhoid bacilli (in contradistinction to the killed bacilli employed by Wright and Russell) in 750 persons, in whom he found in innocuous.

Major Russell in a report in May, 1914, says, "The conclusion is inevitable that the prophylactic vaccine as used in the army has given almost absolute protection against typhoid fever, without producing untoward effects of any character, showing definitely that the vaccination is both efficient and harmless." Those who make the point of their criticism the failure of vaccine to confer absolute immunity, despite the exposure of some patients to exceptionally large doses of typhoid bacilli, or debilitation which has made the body's resistance to the organism ineffective, are unrelenting and unreasonable judges who will be satisfied with nothing less than perfection. Aside from personal idiosyncrasies which may determine failure to produce antibodies, it must be conceded that exhaustion, or the implantation and growth of a virulent strain of typhoid bacilli may have gained too great a headway to be offset by one or more immunizing doses of vaccine. Even small-pox vaccination, the brilliant achievements of which are accepted by scientists, occasionally fails to confer immunity. Likewise, the administration of diphtheria antitoxin may be deferred so long that the body of the patient is overwhelmed by toxins, and no amount of this effective agent can neutralize the effects of the poison; or if an antecedent exhausting illness makes the body a ready prey to the virulent diphtheria toxins, it outstrips the neutralizing antitoxin, and the patient succumbs. And yet, though not infallible, diphtheria antitoxin, and possibly, too, the prophylactic use of tetanus antitoxin and antirabic treatment, will continue as our most potent weapons in those diseases. There is scarcely a potent therapeutic measure that has not its limitations or contra-indications.

### CLASS 4. FAILURE OF IMMUNITY.

This class embraces the cases in which a course of immunizing treatment has been completed, and in which the patients are, nevertheless, after a relatively short interval brought down with an attack of typhoid fever. Here opponents of this measure have found a fertile field for criticism. And it would seem to those not familiar with the literature of the subject that in this particular a significant and incontrovertible objection to immunization has been advanced.

CASE 8.—Among a group of cases that developed in October, 1913, in a neighborhood on the lower east side, where infection had been spread through the milk supply, there were four cases in an Italian family, affecting the mother, a Mrs. C., and three of her children. Through the visits of two married daughters and their children, three additional cases developed in their respective families. A third married daughter, a Mrs. L., whose history illustrates the theme under consideration, also visited quite frequently but was prevailed on to accept prophylactic treatment. Accordingly, two doses of 500 million (killed typhoid) bacilli each, were administered, on October 15 and 25 respectively. November 6, 1913, a final dose of one billion bacilli was injected. These were administered by the Department of Health representatives. All went well with Mrs. L. and her family until March, 1914, when her four children, each in turn came down with typhoid fever, in the following order: Rocco, aged 10, March 10; Theresa, aged 4, March 19; Louis, aged 8, March 19, and Daniel, aged 12, March 29. Subsequent examination of the stools of the grandmother, Mrs. C., at the Research Laboratory, showed typhoid bacilli. She had been the carrier to whom the infection of these cases was traced.

Mrs. L., aged 30, who was about three and a half months pregnant at this time, nevertheless devoted herself assiduously for a number of days to nursing her children and atending to household duties at the same time, until the children were sent to Bellevue Hospital. Despite the fatigue and weakness induced by her overexertions, she continued daily to visit the children at the hospital. (The children ultimately recovered).

April 1, 1914, subjective symptoms of marked lassitude and headache appeared. These Mrs. L. attributed to her pregnancy and gave them no further heed, although they steadily became worse during the second week. At this time sharp cramps developed in the right lower quadrant of the abdomen and persisted despite active catharsis. For four days prior to April 21, her headache, fever, abdominal pain and vomiting grew constantly worse. April 21 she was admitted to the service of Dr. W. Gilman Thompson of the Second Medical Division of Bellevue Hospital. The Widal was positive and a blood count showed 5,000 leukocytes (polymorphonuclears 78 per cent). The spleen was enlarged and an atypical roseolar eruption was present. April 24, at about the fifth month of gestation, she miscarried, as pregnant typhoid fever patients so often do, but this had no influence on the temperature or the course of the disease. Dr. Thompson from his clinical study of the case diagnosed it as one of typhoid fever. April 28 she died. It is to be regretted that no blood cultures during life, or from the viscera after death, had been made. At necropsy, characteristic typhoid lesions were found in the bowels. Peyer's patches in the ileum were greatly swollen, indurated, red and showed ulceration; the spleen was considerably enlarged.

The case of Mrs. L. is then one of a series in which a debilitated condition (in this case from pregnancy and the worry and fatigue incident to nursing her three children affected with typhoid) together with a prolonged exposure to exceptionally large doses of typhoid bacilli overwhelmed the defensive powers of the individual and allowed the infective agent to become dominant.

Case 9.—Miss E. G., aged 29, a nurse doing settlement work on the lower east side of the city, was immunized in June, 1913, and though frail of body worked very hard, nursing, among many others, several typhoid fever patients. In April, 1914, she again nursed several patients of typhoid fever, devoting herself with great energy to their care. April 30, 1914—nearly one year after immunization—she noted definitely the onset of malaise, headache and general pains. May 5 she entered the Post-Graduate Hospital with marked febrile symptoms. May 6 a Widal was negative, but a blood culture showed typhoid bacilli. May 11 the Widal was found positive in a dilution of 1:80 (no higher dilutions were attempted). In the meanwhile her temperature at the beginning of the second week showed the typical continuous elevation, varying only between 104 and 105 F. May 20 she died from intestinal hemorrhage.

Case 10.—Miss D., aged 23, a nurse at the Presbyterian Hospital, received three successive doses of antityphoid vaccine prepared at the hospital, July 1, 11 and 22, 1913, respectively. After nursing a particularly severe case she became ill April 27, 1914, with fever, headache, pains in the back and limbs, and sore throat—that is, nine months after immunization. She continued at her work for several days, despite the indisposition caused by the initial symptoms. On May 2, the Widal test was found suggestive in dilution of 1:20, and negative in dilution of 1:100. Again, May 7, the Widal was positive in a dilution of 1:20, and suggestive in 1:100. May 13, typhoid bacilli were found in the blood. Her temperature curve was an atypical one showing fluctuation in the second week between 98.8 and 103.5, reaching 105 at the end of the fourth week, and then continuing its large excursions. After a stormy period of eight weeks she recovered.

Again one turns to the record of Russell's experience, and there one finds a telling and satisfying answer in quotations from his writings already given, where he says that "by all possible laboratory tests, the immunity conferred was identical with and equal to that remaining after typhoid fever."

To place the problem here presented in different words: If, notwithstanding an attack of typhoid fever, thoroughly authenticated by laboratory and clinical diagnostic tests, there still may follow a second attack of the disease at some later period, even though it is commonly taught and accepted that one attack confers immunity for life, then why should immunity obtained by vaccination be deprecated and tabooed if a like exceptional recurrence follow its practice? That vaccination gives immunity to great masses of those exposed, is most eloquently attested to by the vigorous and severe test it has received in our army and navy among more than 200,000 men. And this should outweigh overwhelmingly the criticism that an occasional exception has evoked. From the literature we shall first cite the reports of recurrences of typhoid fever, and then compare them with the reports of typhoid fever cases subsequent to vaccination, remembering that it is currently held that the latter confers immunity lasting from two to two and one-half years, according to Firth of England. Martha Wollstein, it may here be observed, is quoted as believing in revaccination after one year, though this is contrary to Firth's and Russell's belief in a two or two and one-half years' immunity as noted in their extensive experience in India and the United States, respectively.

1. Some reports of recurrences are as follows:

Dreschfield in over 2,000 cases at the Hamburg General Hospital reports that fourteen were affected twice, and one three times.

In 500 of Osler's cases in which special inquiry was made as to the history of a previous attack, eleven, or 2.2 per cent, were found in which it had occurred.

W. T. Cummins and P. K. Brown found that of 149 of their cases nine had relapses, one of them occurring four weeks after the patient left the hospital; one other patient developed a second attack after six years.

As to relapses, Strumpell reports that they occurred in 9 per cent of all cases of typhoid fever at Leipsic; Murchison, 3 per cent; Griesniger, 6 per cent; and Shattuck as high as 16 per cent. McCrae in 28,057 collected cases found 2,493 (8.8 per cent); while in 172 of his own cases relapses occurred in 11.4 per cent. Why these relapses should occur is a question at once difficult and not in place here to consider, except to indicate that Durham's explanation of the evolution and unexpected dominance of a heretofore subordinate and quiescent strain or family of Bacillus typhosus is quoted with approval by McCrae.

2. Directing our attention to the occurrence of typhoid after a complete course of immunization, we find the following instances recorded in the literature:

Russell reported twenty-seven cases of typhoid occurring among the men of our army and navy, with one death, the result of intestinal hemorrhage. Of these twenty-seven cases of infection in vaccinated persons four cases developed in less than one month after vaccination; eight cases developed within three months after vaccination; six cases developed within six months, and over three months after vaccination; six cases developed within twelve months, and over six months after vaccination; two cases developed within eighteen months, and over twelve months after vaccination; two cases developed within twenty four months, and over eighteen months after vaccination; in three cases the interval was unknown.

Here again, the relatively long period elapsing between the time of vaccination and the appearance of infection would argue powerfully against the theory of a negative phase, if further argument were needed.

Hunt, as already mentioned in a different connection, among 761 who were vaccinated, found two in whom typhoid fever followed the third injection.

In the Massachusetts General Hospital, 1,361 were vaccinated, and but two cases of enteric fever developed (a morbidity of 0.15 per cent) against eight cases in 674 non-immunized persons (a morbidity of 1.19 per cent). At this institution, of the two cases that developed, both were in nurses, one of whom a year after inoculation cared for two patients outside the hospital, and developed a severe attack, but recovered. The other nurse was free two years after vaccination, but then became infected from typhoid material that accidentally entered her mouth.

Here, then, to summarize the results of this review of the literature, are facts obtained from two different angles, which, brought to a focus, show distinctly that whether it is by an attack of typhoid fever, or by the prophylactic injection of killed typhoid organisms, the immunity acquired by great masses of individuals can be safely depended on, in the one case for a protracted period or even a lifetime, in the other case for a period of from two to two and one-half years or even longer.

When it is seen that in those inoculated, a great decrease in morbidity and mortality as contrasted with uninoculated groups of a population results, then indeed is enthusiastic belief in its value justified. Experiences many times multiplied, like those of Wright at Ladysmith, confirm one's faith in its protective power. Of 1,705 persons whom he inoculated, only 2 per cent contracted typhoid as against 14 per cent among 10,529 uninoculated persons; and again elsewhere, among 19,069 inoculated, 266 cases of typhoid (1 in 84.4 persons) developed with a mortality of 17 per cent, while among 150,231, 3,739 cases (1 in 40 persons) developed with a mortality of 25 per cent.

No better statement of the present day attitude on the subject of the possibility of obtaining absolute immunity through antityphoid prophylactic treatment could be given than is contained in a letter of Major Russell written from Vera Cruz to the Department of Health, August 10, 1914, and received after this article had been written. We here take the liberty of inserting quotations from it:

As you will note (in the article written in June, 1912, in the American Journal of the Medical Sciences), there was one fatality among the 27 cases, and that from hemorrhage.

Twenty of the 27 cases occurred within one year of vaccination, probably because of the greater exposure of recruits to all kinds of infection than occurs among older soldiers. It is evident that the vaccine cannot be expected to give absolute protection, even in the period immediately following it. To be sure, it does protect in almost every instance, but the exceptions Many solutions of the problem have been offered, but so far, are frequent enough to prevent our claiming absolute protection. There is no perfectly satisfactory explanation of the occasional failure to protect. We know, of course, that clinical typhoid is not infrequently followed by relapse, and that the disease may occur twice in the same person. It may be due to an excessive dose of infectious material, as in milk epidemics, or among nurses, or to the apparent impossibility of obtaining a high degree of immunity in certain persons.

I do not believe we are justified in promising absolute immunity even for a short period; we can state, however, that the chances of infection among vaccinated persons is extremely small and that when infection does occur, the prognosis is decidedly better than among the unvaccinated (1 death in 27 cases).

We feel very confident that the vaccine protects against the average dose of infectious material, but we have always doubted its power to protect against excessive amounts. While it is true that the protection conferred is not absolute, that is no reason for not using it in dispensaries, hospitals and among contacts as much as possible.

Not a single case of typhoid fever has occurred among the troops with whom I am serving, since they were first mobilized in Texas over two years ago; we are not exposed, however, to milk epidemics to any great extent, since the use of canned milk among troops, while in the field, is almost universal.

It is earnestly desired that all experiences of private physicians in this community, along the lines above indicated, be sent to the department that it may co-operate in their investigation and proper classification, with a view to correlating the mass of testimony to serve for future guidance.

The experiences of the New York Department of Health as here offered indicate that in a civic community no conclusive or valuable study, such as of prophylactic antityphoid injections, is possible, unless the Department of Health can serve as a clearing house to which the facts of isolated experiences of individual physicians are sent, and through which they pass as a well-ordered, logically arranged whole.

GENERAL CONCLUSIONS.

1. The accurate observations recorded in hundreds of thousands of cases leave no doubt as to the preventive powers of antityphoid vaccination in all but a relatively insignificant few.

2. In those subsequently affected it strikingly decreases the morbidity and the mortality.

3. Severe reactions, if one makes observations from extensive studies (the only correct way), are rare.

4. To avoid severe reactions one must observe carefully several precautions, as follows:

(a) Never administer it to any but the healthy.

(b) To permit of slow absorption, avoid puncture of a vein, or intramuscular injection.

(c) Clean syringe and sterilize the area for injection, using tincture of iodin for the latter purpose.

(d) Children especially are to avoid exposure to the sun following treatment.

(e) Avoid administering it during the menses or pregnancy.

(f) Allow no hard work or indulgence in alcohol after the injection.

(g) Avoid reinjecting in indurated areas.

5. Severe reactions have never left permanent injury.

6. When the incubation period has begun, the time for antityphoid immunization has passed. The treatment is preventive of typhoid fever, and not a typhoid antitoxin.

7. Long exposure to overwhelming doses of typhoid bacilli (in those who are in close contact with cases and especially in epidemics), may nullify the immunizing powers of antityphoid vaccine, and an attack may therefore follow one or more injections.

8. Chronic illness, (tuberculosis, etc.,) as well as debility from other causes, and fatigue and exhaustion as well, predisposes to severe reactions.

9. Injections after intimate and long exposure hasten the onset.

10. For a period of at least two years, and possibly more, immunization is as effective in protecting from an attack of typhoid fever as is a previous attack of the disease itself.

11. Recurrences may follow after a complete immunizing course of treatment, in exceptional instances in which debility and fatigue exhaust the resistant and defensive powers of the body, and when exposure to massive doses of typhoid bacilli exists.

Thankful acknowledgment is made of suggestions and assistance received from Dr. John S. Billings, director of the Bureau of Infectious Diseases of the Department of Health.—Journal American Medical Association.

1855 Seventh Avenue.

---

Scranton, Pa., February 24, 1915.

Atlanta Journal Record of Medicine: You may possibly be aware that for the last five years this Commission has been working as actively as seemed feasible on the question of educating the people and arousing more interest among physicians on the cancer problem. The object being, of course, to call attention to early warning symptoms, and the necessity for treatment with no delay and hence the reduction of cancer mortality.

V.

All our work seems to indicate to us that, at the present time at least, the most necessary thing by far is to improve the attitude of the medical profession towards cancer. Statistics that we have recently collected show that, in this State at least, the physician has his cancer cases under observation for an average of over one year before resorting to radical treatment. Undoubtedly many lives are sacrificed in this country every year by this policy.

In order to overcome this condition we have devised the following plan in which we most urgently request your co-operation. Our plan is as follows: We hope to induce every medical journal in this country to publish a special cancer number simultaneously. For the monthly journals this is to be for the July issue; for the weeklies this is for the first issue in July. It is not necessary that all the original articles be devoted to cancer, but we hope that at least three or four articles may be. Second, we hope that each journal will contribute one or more strong editorials on this cancer campaign, its aims, importance, etc. Third, in order to call special attention to the subject we are asking each journal to contribute a full page advertisement as enclosed. We wish to say at once that the last paragraph is entirely at the pleasure of the various editors. We in no way desire its insertion for ourselves but we thought some editors might wish to include it as an explanation of the movement. We earnestly hope that your valued journal will join this plan.

We feel that if we can secure the co-operation of all our American journals that it will mean the greatest result that has ever been accomplished in this connection in any country. We believe too that such a movement will redound very much to the credit of American Medical Journalism both in this country and abroad.

We have already obtained full co-operation in this matter by the New York Medical Journal, The Medical Record, The American Journal of the Medical Sciences, Surgery, Gynecology and Obstetrics and the Pennsylvania Medical Journal. Dr. W. L. Rodman, not as one of our members, but as President of the American Medical Association, is already preparing two articles for our cancer numbers.

We may say too that as a subsidary part of this plan we are writing to every county society in the country to try to induce them to hold a cancer symposium in June. We feel that cancer symposiums all over the country in June to be followed by a flood of cancer literature the following month will have an effect which will last for years. We believe that it will place the American medical profession above reproach in this detail and above all that it will lead to the saving of hundreds of lives by inducing earlier radical treatment.

We sincerely hope that you will give your cordial aid to this plan and join the movement with your Journal in July. We will much appreciate an early favorable statement from you on the matter as every journal that comes in will furnish a valuable argument to those who hesitate.

We hope that you will command any of our Committee if we can be of assistance and with assurances of our marked consideration, we remain, Yours very truly,

The Commission on Cancer of the Medical Society of the State of Pennsylvania.

J. M. Wainwright, Secretary.

---

### ANOCI-ASSOCIATION IN STOMACH AND GALL TRACT SURGERY.

By George W. Crile, M. D., F. A. C. S., Cleveland, Ohio.

That the liver is a vital organ is proved by the fact that after excision of the liver animals undergo a loss of energy, indicated by a progressive loss of muscular power and fall of body temperature until death, which usually occurs within twenty-four hours.

Every impairment of the function of the liver, therefore, may not only seriously handicap the patient but may prove a serious menace to life itself. We propose in this paper to point out the possibility that the pathologic states requiring operation on the stomach or biliary passages; or the effect of the operation and the anesthetic; or the combination of these factors may so impair the liver function as to jeopardize or even kill the patient.

Every surgeon probably has performed apparently successful operations on the stomach and biliary passages of patients weakened by disease, by obstruction or by starvation. At the close of the operation the prognosis may seem promising. but the following post-operative picture soon begins to develop: The patient becomes increasingly apathetic; his energy is slowly depleted; though tired and weak, he cannot sleep; his tongue becomes increasingly dry; the pulse gradually rises and its volume steadily decreases; bile is suppressed; nourishment is refused; the patient's demeanor is cheerless and despondent. The patient seems to be steadily sinking in the quicksand of some fundamental failure or normal metabolism. No means suffice to change the course of events. The patient either gradually recovers or else he gradually becomes first delirious, and then unconscious until death.

This is the familiar picture after operations on patients starved because of pyloric obstruction, or because of protracted vomiting from any cause; or after operations on patients exhausted by chronic empyema of the gall-bladder or by long-continued infections from common duct stones. The temperature and pulse of these starved patients may be normal. If the prognosis be based on pulse and temperature alone, therefore, the greatest errors may be made.

I did not realize the full significance of the fundamental metabolic conditions in these cases before I made use of the transfusion of blood in the case of a patient in an advanced stage of starvation resulting from pyloric obstruction. Blood transfusion carried this patient through the operation with an efficient circulation, with pink lips even, which was maintained after the operation also. But the early promise of success disappeared as the metabolic obstruction caused the picture to change. In spite of the good circulation the patient declined through increasing apathy and progressive somnolence to unconsciousness and death.

I have seen this metabolic quicksand inaugurate the same fatal sequence after long delayed operations upon emaciated, starved common-duct cases, especially when the duct was packed with stones; and after the most careful explorations in advanced cases of cancer of the stomach.

Happily the fatal sequel is rare, but many patients having wider margins of safety sail perilously near the rocks.

It seemed to me of vital importance to find the explanation of these phenomena; and to discover by what means the slender margin of safety in these cases may be preserved or perchance widened.

The key to both problems was furnished by certain experiments in progress in my laboratory which were being carried on to discover what tissues and organs of the body were concerned in the transformation of the energy acquired from the environment into energy available for the needs of the body. In these experiments histologic examinations were made of every organ and tissue in animals which had been subjected to prolonged insomnia; to infection; to fear; to physical injury under anesthesia. In every case histologic changes were noted in three organs only, the brain, the suprarenals and the liver. In each of these organs the cells become vacuolated and shrunken, the nuclei misshapen and some nuclei disappeared. In proportion to the intensity of the stimulus and the length of time the stimulus was applied, the number of altered cells and the degree of disintegration increased. In the case of the liver, after prolonged stimulation, the cells stained poorly, the cytoplasm was vacuolated, the nuclei were crenated, and the cell membranes were irregular, the most marked changes occurring in the cells nearest the periphery of the lobules.

That the histologic changes in the liver are not due to metabolism or to toxic products but that they are "work" changes incident to the conversion of latent into kinetic energy and that the brain, the suprarenals, and the liver are interdependent is proved by the facts (1) that the average duration of life after excision of the liver is about the same as after adrenalectomy —approximately eighteen hours; and (2) that the amount of glycogen in the liver was diminished in those experiments in which there was evidence of brain-suprarenal activity.

From these premises we must consider that the brain, the suprarenals and the liver are mutually dependent upon each other in the conversion of latent into kinetic energy.

Prominent among the products of this transformation of latent into kinetic energy are acids. We may say that every motion, every injury, every physical exertion, every degree of fever, every reaction to infection or to autointoxication, every respiratory movement, every heart beat, produces an acid. Under normal conditions these acids are neutralized into harmless compounds which are eliminated by the kidney, so that under normal conditions the body tissues and fluids are for the most part slightly alkaline. If every activity of the body produces acidity in a greater or less degree, it is vitally necessary for the body to maintain a large margin of safety against acidosis by the presence of alkaline salts and bases which are derived from food. Experiments have shown that for the maintenance of its normal state of slight alkalinity, the body is dependent primarily upon the liver and secondarily upon the suprarenals. When the liver is excised the blood soon loses its slight alkalinity and in a few hours becomes acid. When the suprarenals are excised the alkalinity of the blood is maintained for a longer period—perhaps twice as long—but it then becomes acid, and in each case the acidity of the blood is the close precursor of death. The excision of no other organ in the body produces this tendency to immediate acidosis.

We can not dwell here upon other experimental observations which show the relative activities of the liver and the suprarenals in this neutralizing process, nor the additional evidence that the liver and the adrenals are directly controlled by the brain, which also controls the transformation of energy, which in turn, as we have already stated, always produces acidity; we will merely recapitulate by saying that the harder the body is driven by any stimulus, the more rapidly will latent energy be transformed into kinetic energy. The more rapid the transformation of energy—the greater the production of acid. The greater the production of acid, the greater also the strain upon the power of neutralization possessed by the liver and the suprarenals, and the greater the drain upon the body's store of alkalies and bases. When the liver and the suprarenals are overtaxed and the alkalies and bases are exhausted, the state of acidosis is reached.

Still another fact established by laboratory experimentation has an important bearing upon our problem—especially as it applies to common duct operations. The liver performs its function in part through hormone action and in part through direct innervation. It is curious that for the performance of at least a part of its function it is necessary for the liver to have a simultaneous hormone and nerve stimulation. Now the nerve supply of the liver is derived from the sympathetic system, the nerve fibers passing along the blood vessels and the common duct. As during the processes of evolution these nerves have been thus abundantly sheltered against injury, they have not evolved physical qualities for their protection as have the peripheral nerves. In the course of common duct operations performed by an operator who is unaware of this grave danger, the nerve supply to the liver will be more or less blocked by the trauma of the operation. If the block be light and the patient have sufficient endurance, the temporary loss of liver function will be safely bridged: on the other hand, the more severe the trauma of the nerves, the more completely will the nerves be blocked and the longer will that block last. This conclusion corresponds exactly with our clinical facts and makes it evident that surgery has been riding rough-shod over an extremely vulnerable mechanism.

Another factor which has a vital bearing upon the treatment of the class of patients we are considering is the anesthetic. Clinically it has long been recognized that when a patient is in a state of exhaustion resulting from infection, from injury, from shock, from starvation, from hemorrhage—from any cause whatsoever—he may never recover consciousness after the administration of a general anesthetic. In a Hungarian reference, the title of which I am, at the moment, unable to find, it is shown that starved dogs inevitably die after inhalation anesthesia. Clinicians know well how unsafe it is to give a general anesthetic of any kind to a patient on the verge of acidosis. A patient with chronic vomiting, with or without chronic pyloric obstruction, with an acetone odor of the breath, with peculiarly pink lips and dry tongue and mouth will, in all probability, never again regain consciousness after being anesthetized. The aged not infrequently die under even a short anesthesia.

Why do not these patients recover? If the patient had the power of consciousness before the anesthetic is administered what happened during the anesthesia to make it impossible for the patient to regain consciousness after anesthesia?

We have already referred to the acid-producing power of stimuli. Shall we conclude therefore that the trauma of the operation alone may have pushed beyond the margin of safety the neutralizing powers of the body already taxed by pre-existing conditions; or is the anesthetic itself a factor in producing the fatal result?

To answer this question Dr. Menten, in my laboratory, made for me observations of the H-ion concentration of the blood under various conditions—the H-ion concentration being an index of the acidity of the blood.

H-ion concentration tests made after the application of many kinds of stimuli, the results of which confirmed the postulate which we have already stated—that acidity is the result of the activation of the body by any adequate stimulus. The blood was then tested to determine the H-ion concentration in ether anesthesia, in nitrous oxid anesthesia, and after the administration of alcohol, and strychnine and of morphin. Both ether and nitrous oxid produced a marked increase in the H-ion concentration, that is, both produced acidity in the blood. After coming out from the anesthetic this acidity was neutralized by the animal in about thirty minues. The lighter the anesthesia, however, the shorter was the period before neutralization was accomplished. This result gave us our clue to the tendency to acidosis and to death under anesthesia of weak and emaciated patients. The increased acidity produced by the anesthesia was sufficient to overcome the already narrow margin of safety. That acid intoxication follows the administration of ether and chloroform has been noted by many observers, that acidity being evidenced by the early appearance in the urine of acetone and later of diacetic acid. It has also been noted, as one writer states, that the "starvation preceding and following the operation is also a factor of considerable importance."

Our experiments have shown, however, that the increased acidity actually develops during the anesthesia itself, sometimes to a fatal degree, and that a starved condition is not only of "considerable" but of prime importance, since it means that the acid-neutralizing power of the liver has been surely impaired —possibly lost.

Two more important clues were obtained from the result of the H-ion concentration tests after the administration of morphine and alcohol. Alcohol caused acidity, the acidity not being so marked, however, as that produced by the anesthetics. The H-ion concentration was not altered by morphine, no matter what the size of the dose. When the administration of morphine preceded the induction of anesthesia then a smaller amount of the anesthetic was required to produce complete anesthesia and the H-ion concentration test showed that the acidity was markedly less than in anesthetized animals which had not received the preliminary dose. The preliminary dose of morphia not only lessened the degree of acidity produced by the anesthetic, but it in no way interfered with the return of the blood to its normal alkalinity; on the contrary—and the following observation is of great significance—if the morphine was given after acidity had been produced by the anesthetic, it postponed the time of neutralization and if given in large doses prevented the animal from overcoming the acidosis. That is, it would appear that morphine controls the mechanism which governs the neutralization or alkalinization of the blood.

In these facts we may find the explanation of the outcome we have described as the occasional sequence, in handicapped patients, of operations on the gall-bladder and stomach. If under normal conditions a definite amount of energy is stored in each of these vital organs: the brain, the suprarenals and the liver, then any reduction of this amount by the psychic or physical environment incident to an operation diminishes by so much the safety of the operation. This is true of any operation, but in the operations which we are considering, when one of these vital organs has itself been attacked by the pathological condition for which relief is sought, the integfirity of that organ is still further endangered.

In this explanation we may find the clue to the solution of our second problem—the development of measures which will protect or perchance widen the patient's margin of safety.

Every further drain upon the energy remaining in the organs of the kinetic system must be minimized or prevented, if possible. In suitable cases pre-operative emotion must be minimized by pre-operative medication. Nitrous-oxid-oxygen instead of ether should be the anesthetic, since this anesthetic does not impair the immunity as does ether.

Nerve blocking with novocain should precede every division of nerve-bearing tissue; trauma should be minimized by employing sharp bloodless dissection, by the avoidance of pulling and tearing manipulations and by employing the least possible amount of sponging. Because of the very slight insulation of the splanchnic nerves, their injury is surprisingly easy; for this reason also, therefore, the two-stage operation is advisable in critical cases, as thus a smaller number of splanchnic nerves will be injured at one time and the interference with the functions of the already almost overwhelmed liver and suprarenals will be diminished. Post-operative pain should be minimized by quinine and urea hydrochloride injected at a distance from the line of incision but including the entire area of operation; the post-operative environment should be made cheerful and hopeful, narcotics benig used when required to prevent pain and undue psychic strain.

In brief, then, the mortality and morbidity attending gastric and gall-bladder operations may be still further reduced by using methods which minimize trauma, pre-operative and post-operative emotion and pain and by so doing conserve the energy remaining in the cells of the brain, the suprarenals, and the liver in patients in whom the stores of energy in their kinetic organs have already become depleted by the exhaustion due to the disease from which they seek relief.

To recapitulate—for the class of patients we have been considering—those handicapped by the exhaustion which is incident to pathological conditions of the liver and stomach, the ideal treatment comprises:

1. The pre-operative administration of sodium bicarbonate and glucose and of bromide per rectum. Morphine is contraindicated if acidosis is present or threatened.

2. Eether twilight anesthesia or a light nitrous-oxid-oxygen anesthesia.

3. A technique so accurate and so completely associated by the use of local anesthetics and gentle manipulations that but a small amount of the anesthetic is needed.

4. As rapid a technique as is consistent with good work that the period of anesthesia may be as short as possible.

The clinical record upon which these conclusions are based include the histories of 893 operations on the biliary tract and 331 on the stomach performed by my associates, Dr. Bunts and Dr. Lower, by the members of my surgical staff at the Lakeside Hospital and by myself.

DISCUSSION.

Dr. H. M. Richter (Chicago): Some time ago, when I was told I was to have the honor of opening the discussion on Dr. Crile's paper, I went to Cleveland with a view of seeing some of his work, and at Dr. Crile's very kind invitation the other day I had a chance to watch his work, and I want to say that it is hard to discuss his paper andcrit icise it after seeing his work. Anyone who has seen his surgical technique, and compares it with the technique seen in the ordinary clinic of the country realizes that there is no room for comparison. To see a patient with a weak place in his groin converted into a really dangerously sick individual, who needs a special nurse to restrain him for some hours or a day or two after having a few sutures placed to close up such an opening must strike one as illogical. It does not seem fair to the patient. If we were to regard such a patient from a viewpoint of the examiner for life insurance, we would decide that the patient's case in a few minutes, perhaps converting the case from a fair risk into a poor one. If the average cabinet maker converted a piece of good mahogany into a bad piece of wood in a short time, as the surgeon does with a moderately serious lesion like a hernia, he would soon be out of a job. I am quite sure that we misled ourselves from seeing patients day after day being made quite sick by operation. We deceive ourselves in making a patient sick in order to do an operation of the ordinary type, but we are pleased when the outcome is favorable.

I had an idea that Dr. Crile was going to talk on the technique of his anoci-association work, and in thinking it over it struck me that probably the essential elements of his work are his anesthetic and local blocking, his morphinization, and last, but not least, the general atmosphere with which the patient is surrounded. It is rather hard to criticise, even constructively, work which is really so beautiful, and yet I was impressed that some of the phases of his work are not essential to the end gained. I mean the end gained by Dr. Crile is due largely to one or two or possibly three factors. It struck me the nitrous oxid anesthesia as given in the operating room is quite different from the nitrous oxid anesthesia as we understand it. We understand the nitrous oxid type of anesthesia to be that by which we hold the patient down while being operated on, but in Dr. Crile's work that does not apply. The patient is fully relaxed, and is asleep, and when I called attention to the point of allowing the patient to remain asleep quite a few minutes longer than necessary to do what he intended to do, Dr. Crile reminded me that a patient asleep was comfortable. I really think these are factors in the results obtained by him—his accurate surgical technique, the lack of damage done to the tissues, and the nitrous oxid anesthesia.

Local blocking impressed me as being a valuable thing, but I must say that in some of our nitrou oxid work blocking really is not the whole factor in the patient awaking without local pain or without distress afterwards. Under nitrous oxid anesthesia a patient undergoing an operation of moderate magnitude, as an operation for hernia or an appendix operation, should come out from under the influence of the anesthetic without any local pain. As a matter of fact, in my own hernia work, where I use nitrous oxid, I usually allow the patients to get up at once after operation, figuring that the sutures will hold quite as well the first day as the tenth day, and they usually do not complain of pain. There is no subconscious memory or any other factor at work. My technique is perhaps rough as compared with that of Dr. Crile. I remove the vas from the sac by means of dry gauze and ligate the peritoneum at the neck of the sac in the usual manner. While these patients are weak a minute or two after the operation is over, they really complain of no pain.

The same applies to major amputations. I have failed to see any material effect after major amputations from the blocking of nerve trunks. In major amputations performed under nitrous oxid anesthesia, and with moderate speed in one's work, it seems to me the patients do not complain of post-operative pain that would be weakening and depressing. Most of the pain complained of was in a case in which I injected novocain before anesthetizing the patient, and when there was freedom from pain, I could wiggle his leg, in spite of the fact that amputation was done for a crushing injury of the middle of the ithgh bone. That patient on awakening from the anesthetic was free from pain for an hour or so, and then he complained of excruciating pain for many hours.

I wish Dr. Crile would explain the object of blocking in his stomach and gall-bladder work. I think it is the general experience of men who have done any abdominal work under local anesthesia, that aside from dragging and pulling, the abdominal viscera are not sensitive to pain in the ordinary sense. They are not sensitive to a common sensation at least. They will stand any amount of cutting or suturing, provided you do not work dow down and interfere with the reflections of the posterior peritoneum. In my own experience I find I can do stomach work under local anesthesia. I have done gastro-enterostomies. In advanced cases of cancer of the gullet I have done the Tom operation. I have drained the gall-bladder under peculiar conditions, and I have cut annular strictures at will, provided I did not make pressure on them and if one uses Dr. Crile's technique in doing a gastro-enterostomy or a gall-bladder operation, it seems to me local blocking would be of little need.

I do not know about the effect of blocking upon the common duct, but of the gall-bladder I can speak. I am quite sure a patient is insensitive to any amount of suturing, to the placing of a rubber tube in the gall-bladder, to doing gastrostomy, and doing an extensive Tom operation.

As to acidosis, I can not speak as to the way in which anoci acts. I do not see the relationship between the acid reaction of the body, and as Dr. Crile expressed to me, the acid reaction of the battery. The acid may apply to both.

Regarding the pictures which were shown, he did not mention one fact which is so important that it ought to be referred to, although I am sure staining of the sections was done under proper conditions. These double sections were all stained on the same slide and under the same conditions, so that if the various depressing agents Dr. Crile enumerated are the cause of the digerence in staining, this digerence may be taken for granted. But the difference in staining is there; it is not artificial.

Dr. C. D. Schaeffer (Allentown, Pa.): As only a few minutes are allotted for discussion, I desire to confine my remarks more or less to what was said by the first speaker in reference to common duct obstructions. He has indicated that we have still a high mortality, namely, a mortality of 90 per cent. At the present time there are many operators who have a mortality of 23 per cent. This high mortality, I believe, is due largely to manipulation, inasmuch as this occurs in very fat individuals.

In reference to improvement in the technique, I desire to submit to you a little instrument which I have been using for the last four years, and which I am satisfied simplifies the procedure and diminishes the traumatism inflicted. This instrument consists of a blade which is closed on one side. It is passed in the foramen of Winslow posterior to the stone, and after locating the stone it is closed just as tight as possible, and the stone brought up into the opening. If you are not sure the common duct is patulous from there on out, you should introduce a suture on each side before incising the duct for the removal of the stone. These two sutures on either side are then held by an assistant, and an incision is made, and the stone extracted. After the extraction of the stone this can be opened and the duct explored above and below.

I have used this instrument in twenty-three cases with uniform results, and was prompted to do this at the suggestion of Dr. Crile. I note in his paper he suggested that closure of the common duct is preferable to introducing a drainage tube, and I have followed it with surprisingly good results. There was no leakage until the drainage tube was removed. We are convinced of that. We introduced to the point of incision a small cigarette drain, and this was removed at the same time we removed the drainage tube.

Dr. Walter Hamburger (Chicago): In connection with Dr. Crile's paper, I wish to say that Allen, of the Rockefeller Institute of Medical Research, has recently done some work on diabetes which shows, in addition to neutralizing the acids in diabetic acidosis, the value of alcohol as a combustible fuel and that it was a better method. Instead of post-acidosis with neutral soda, a greater result might be obtained by burning up by alcohol. In accordance with the work and suggestion made by Dr. Billings, I recently had the opportunity to try the method in two operations on diabetics, where the margin of safety is still lower because of the post-operative coma which ensues and because of the preceding acidosis as a part of the diabetes. In one of these patients where we suspected a tumor in the pancras itself, in addition to using a modified anoci technique we gave post-operative fifteen per cent alcohol enema and glucose, and the result was splendid. Instead of any acidosis or impending coma, the patient made a recovery. In the other patient who underwent a laparotomy, while the anoci had much to do with the result, we think the alcohol enema drop by drop was of greater value.

I would like to ask Dr. Crile whether that would not still widen the margin of safety he speaks of, in addition to feeding with soda bicarbonate beforehand and using drop by drop enema.

Dr. George W. Crile (closing): I have been much interested in Dr. Richter's work and in what he has said. I will explain briefly the blocking we do in these cases. Dr. Frank, who is present, knows we do not block the intestines or the liver or gall tracts, but only the abdominal wall, and Dr. Richter knows well there are no anoci ceptors in the intestinal area, and we block only for that condition which suggests obstruction of the bowel or intestine.

I desire to express my appreciation of Dr. Hamburger's remarks with reference to alcohol. I have no idea what would be done there. It is an excellent suggestion and one that can be easily worked out. One disquieting feature in doing anoci-association work is that it increases the high concentration of the cell in the body. It burns up the acid, and it must do more good than it does harm, and perhaps both works.—Lancet Clinic.

---

### OBSERVATIONS ON HEADACHE.

By L. Sexton, B. S., M. D., New Orleans, La.

The common forms of headaches are toxemic, uremic, ocular, uterine, neurasthenic, syphilitic, migraine, diabetic, congestive. Causes are reflex, organic and functional, and due to syphilis, malaria, acute and infectious diseases and chemical poisons, uremia and toxemia of pregnancy and rheumatic and gouty conditions. Meningeal conditions, tubercular or syphilitic, cause constant headaches. Evening temperature and other physical signs would incline one to tuberculosis as the cause of the headache. Intra-cranial tumor headaches cause vertigo, vomiting, and later paralysis or softening of the brain. Headaches caused from ear diseases are associated with running discharges, earache, or tenderness upon pressure of the mastoid cells. Frontal-sinus infection, following catarrh, produces continuous pains, also tenderness upon pressure over the sinus. Supra-orbital headache is due to irritation of the trigeminal, or fifth pair of nerves. The nerve seems to be pressed upon at its exit through the orbital foramen. Chronic interstitial nephritis, with only a trace of albumin, specific gravity of urine low, frequent passage of large quantities of urine at night, is often accompanied by headache. There is high blood pressure showing that the vascular, as well as the nervous system, plays a part in chronic headaches. Lithemic or bilious headaches come from renal or hepatic derangements, or choked flues, so to speak, and are relieved by brisk purgation and change and a cut in the amount of food ingested. Headaches due to fever are best relieved by sponging, and giving five-grain doses of some one of the coal-tar group of remedies that relieve headache and reduce fever at the same time.

Mental exhaustion, bad air, ill-ventilated rooms, crowded picture shows, all predispose headaches, which should be prevented by avoidance of such places and conditions. Carbohydrate and nitrogenous diet should be almost excluded in the case of all chronic headache sufferers.

### DIFFERENTIAL DIAGNOSIS.

Emetics and purgatives relieve toxemic varieties of headaches. There is albumin with the uremic variety, plasmodia with malaria. Examine the blood. Malaria headache is also intermittent. Alcoholic headache follows debauch. Reflex or uterine follows female trouble; note history of case. Note Wassermann reaction and history in syphilitic cases. Headaches of arterial or nephritic origin usually occur in high-livers and low thinkers, in patients past the age of fifty, who keep their stomachs full and seminal vesicles empty; cabaret or saloon frequenters, who may clean the sewers at their houses, but neglect the twenty-nine feet within their own person, men who acquire a camel's thirst, but have a natural aversion to water. They occur mostly in the morning after a half, or all, night carousal. "A man is as old as his arteries," these being prematurely hardened by sedentary habits, over-eating or drinking and underworking; day laborers rarely have headaches. Such headaches are relieved by brisk purgation and diuretic alkaline mineral waters, reclining one to two hours midday to take off the heart load, and a reversal of all previous habits of overeating and drinking. Neurasthenic headaches are often due to insomnia and the anemia accompanying the run-down unbalanced condition of the patient, and the strain upon the central nervous system. Post-operative neurasthenia, due to nervous changes incident to the removal of certain glands, or organs, whose secretions are necessary for proper equilibrium of the nervous system, usually have low blood pressure, as neurasthenia is mostly confined to young subjects whose arteries are not hardened. Periodic headaches and the migraines are mostly due to syphilis, malaria, menstrual epochs, congestion, rheumatic and gouty diathesis and climatic changes. Among students and young adults, from ten to thirty-five, astigmatism, myopia, hypermetropia, eye-strain of students and school children and persons doing work requiring constant eye-strain in a bad light is a frequent cause of headaches. Intestinal stasis, or autotoxemia, is a common cause of headaches. Nausea, vomiting, purging or constipation, sallow skin, puffed under the eyes, coated tongue gastroptosis and enteroptosis, unusual prominent abdomen, are the most common symptoms of this variety of headaches.

### PREVENTION AND TREATMENT.

As headache is a symptom and not a disease, we should first ascertain and remove the cause of the trouble. As, for example, in the headache of toxemia of pregnancy, we should open up all the avenues of elimination, thus reducing the blood-pressure and the toxemia caused by the conditions. Diet should be restricted to easily digestible liquid foods, such as buttermilk or skimmed milk, vegetable soups, sea foods, cereals, green vegetables, stewed and ripe fruits, and we should prescribe drinking freely of alkaline mineral waters. Subjects of such headaches should avoid red meats, fried food, all condiments and alcoholic beverages. Free purgation with compound jalap powder, or other mild laxatives, followed by five-grain doses of phenacetine or acetanilid or twenty-grain doses of sodium bromide, repeated an hour or two apart, as required. The patient should retire to a dark, well-ventilated room, where absolute rest and quiet can be secured. The cephalalgia of syphilis can be permanently cured only by prolonged use of constitutional treatment with mercury, iodide of potash or salvarsan (in the early stages of the disease), coupled with proper living and hygienic surroundings. Five grains of acetanilid with half-grain caffein and one grain of monobromate of camphor, acts splendidly in relieving the head and leg pains of syphilis. Five grains of aspirin, or bromide of sodium, may be used as a change from the first-named prescription.

The blood should be examined for plasmodia malaria in remitting types of cephalalgia. If plasmodia are found, five grains each of quinine, salol and phenacetin three or four times daily, after a calomel and soda purge, should relieve the patient. In gouty and rheumatic types, free elimination by alkaline purgatives and diuretics are essential to a cure. Give iodides with wine of colchicum in the gouty, and any of the salicylates freely for the rheumatic types. The drinking of hot water and bathing in hot water or steam baths or with hot packs, are as efficacious at home as at hot springs, and should be stressed in all cases with rheumatic tendencies. For the immediate relief of pain, any of the coal tar group in five-grain doses, with caffein or strychnia if the heart is weak, have been the most useful agents at our disposal.

Headaches from acute indigestion should be relieved by alkaline emetics and purgatives, rather than by hypodermics of morphia, so much in vogue. Mentholated alkaline drinks, lime water or soda, are grateful after the stomach has been emptied of its soured contents. Ocular headaches are best relieved by the proper fitting of suitable glasses, which relieves the eye-strain.

The prevention of all headaches is accomplished by removing the cause of the trouble; for example, children or students with eye-strain headaches should not only have proper glasses fitted, but they should do their studying in the daytime if possible, and if any night work is required they should have the best light suspended over the left shoulder, with the eyes protected by a green shade. Constipation headaches should be relieved by a vegetable diet, fruits, hot water upon rising, abdominal massage or any of the simple laxatives, which should be changed before the bowels require larger doses.

Headaches from any of the neuroses should be relieved by the proper treatment of that particular nervous condition. Nervous cases are often benefitted by active exercise in the open, changes of associates and environments, living among strangers rather than with too-sympathetic relatives. Eating substantial home-cooked food and earning the price of the food by some agreeable occupation, relieves many of the headaches of the idle luxurious class, who exercise so little that they do not eliminate the poisons generated within themselves. People taking plenty of exercise are very rarely troubled with headaches.

Congestive headaches at menstrual periods are prevented by free purgation, hot hip baths, hot-water bags, confinement to dark and well-ventilated rooms the first day of the periods with hot drinks and applications until the flow is established.

Diabetic headaches are prevented by taking plenty of alkaline mineral waters and the avoidance of all sweet and sugar-containing diets.

In the treatment of headaches the same remedies should not be used in successive attacks; the patient should not know what drugs are given, as drug habits are frequently formed by the use of many of the patent medicines and headache mixtures. Headache is the cause of more unreasonable drugging through proprietary and patent medicines than any other disease. We have seen sufferers from the headache of heart disease and edema of the lungs cyanosed from acetanilid (the prominent ingredient of nearly all headache remedies) on account of the cheapness of the drug. The story of the damage and deaths from such treatment is partially told in the numerous sudden deaths reported as heart disease and apoplexy. The Government should force all such preparations to print "Contains acetanilid, a heart depressant and poison," in a prominent place and in large letters on every package and bottle offered for sale to the public.

Hyperacidity of the stomach is a frequent cause of headache. In such cases all food-producing acidity (usually sweets and starches) should be excluded, and lime water, soda or some alkali should be freely administered to neutralize the acidity. This often relieves the headache without the use of other drugs. Many neurotic women, from constipation, lack of nourishment and worry, have what might be called nerve or "brain-storm" headaches. This type often drifts into drug addiction, for which they blame the doctor. Hypodermics of morphine should be used only as a last resort in all such cases. —Medical Council.

